# Effect of *TNF-α *genetic variants and *CCR5*Δ*32 *on the vulnerability to HIV-1 infection and disease progression in Caucasian Spaniards

**DOI:** 10.1186/1471-2350-11-63

**Published:** 2010-04-26

**Authors:** Sergi Veloso, Montserrat Olona, Felipe García, Pere Domingo, Carlos Alonso-Villaverde, Montserrat Broch, Joaquim Peraire, Consuelo Viladés, Montserrat Plana, Enric Pedrol, Miguel López-Dupla, Carmen Aguilar, Mar Gutiérrez, Agathe Leon, Mariona Tasias, Josep Ma Gatell, Cristóbal Richart, Francesc Vidal

**Affiliations:** 1 Hospital Universitari de Tarragona Joan XXIII, Tarragona, Spain; 2 IISPV, Tarragona, Spain; 3 Universitat Rovira i Virgili, Tarragona, Spain; 4 Hospital Clínic, Barcelona, Spain; 5 Hospital de la Santa Creu i Sant Pau, Barcelona, Spain; 6 Universitat Autònoma de Barcelona, Barcelona, Spain; 7 Hospital Universitari de Sant Joan, Reus, Spain; 8 CIBER Fisiopatologia de la Obesidad y Nutrición (CB06/03), Instituto de Salud Carlos III, Madrid, Spain; 9 Hospital de Sant Pau i Santa Tecla, Tarragona, Spain; 10 Universitat de Barcelona, Barcelona, Spain

## Abstract

**Background:**

Tumor necrosis factor alpha (TNF-α) is thought to be involved in the various immunogenetic events that influence HIV-1 infection.

**Methods:**

We aimed to determine whether carriage of the *TNF-α-238G>A, -308G>A *and *-863 C>A *gene promoter single nucleotide polymorphisms (SNP) and the *CCR5Δ32 *variant allele influence the risk of HIV-1 infection and disease progression in Caucasian Spaniards. The study group consisted of 423 individuals. Of these, 239 were uninfected (36 heavily exposed but uninfected [EU] and 203 healthy controls [HC]) and 184 were HIV-1-infected (109 typical progressors [TP] and 75 long-term nonprogressors [LTNP] of over 16 years' duration). *TNF-α *SNP and the *CCR5Δ32 *allele were assessed using PCR-RFLP and automatic sequencing analysis methods on white blood cell DNA. Genotype and allele frequencies were compared using the χ 2 test and the Fisher exact test. Haplotypes were compared by logistic regression analysis.

**Results:**

The distribution of *TNF-α-238G>A, -308G>A *and *-863 C>A *genetic variants was non-significantly different in HIV-1-infected patients compared with uninfected individuals: *-238G>A*, p = 0.7 and p = 0.3; *-308G>A*, p = 0.05 and p = 0.07; *-863 C>A*, p = 0.7 and p = 0.4, for genotype and allele comparisons, respectively. Haplotype analyses, however, indicated that carriers of the haplotype H3 were significantly more common among uninfected subjects (p = 0.04). Among the infected patients, the distribution of the three *TNF-α *genetic variants assessed was non-significantly different between TP and LTNP: *-238G>A*, p = 0.35 and p = 0.7; *-308G>A*, p = 0.7 and p = 0.6: *-863 C>A*, p = 0.2 and p = 0.2, for genotype and allele comparisons, respectively. Haplotype analyses also indicated non-significant associations. Subanalyses in the LTNP subset indicated that the *TNF-α-238A *variant allele was significantly overrepresented in patients who spontaneously controlled plasma viremia compared with those who had a detectable plasma viral load (genotype comparisons, p = 0.02; allele comparisons, p = 0.03). The *CCR5Δ32 *distribution was non-significantly different in HIV-1-infected patients with respect to the uninfected population (p = 0.15 and p = 0.2 for genotype and allele comparisons, respectively) and in LTNP vs TP (p = 0.4 and p = 0.5 for genotype and allele comparisons, respectively).

**Conclusions:**

In our cohort of Caucasian Spaniards, *TNF-α *genetic variants could be involved in the vulnerability to HIV-1 infection. *TNF-α *genetic variants were unrelated to disease progression in infected subjects. The *-238G>A *SNP may modulate the control of viremia in LTNP. Carriage of the *CCR5Δ32 *variant allele had no effect on the risk of infection and disease progression.

## Background

TNF-α is a pleiotropic cytokine that acts as an immune and inflammatory mediator. It is synthesized mainly by macrophages and T-cells and most of its actions take place by binding to two cell receptors: TNF-α R1 and R2 [1]. Investigations suggest that TNF-α is involved in the pathogenesis of HIV-1 infection since it is overproduced by infected individuals [2-4].

Like many other proinflammatory cytokines, the production of TNF-α is at least partially genetically determined [5,6]. Several functional single nucleotide polymorphisms (SNP) have been identified within the *TNF-α *gene cluster [5-8], the most widely studied of which are a *G>A *transition at position *-238*, a *G>A *transition at position -*308*, and a *C>A *transition at position *-863 *[9]. Several cohort studies have assessed the influence of *TNF-α *SNP on the immunogenetics of HIV-1 infection. The information available on how *TNF-α *genetic variants affect vulnerability to infection is inconsistent [10-14]: some studies have found no effect [11,13,14] while others have found a strong association [10,12]. Case-control studies have assessed the influence of *TNF-α *genetic variants on HIV-1 disease progression in infected patients [10-12,14-17]. While some of these studies have reported an association with progression [10-12,16,17], others have failed to find such an association [14,15]. A minority of untreated HIV-1-infected patients show an uncommon clinical form of infection characterized by a non-progressive disease and, sometimes, by self-limited viremia over the years. These patients are known as long-term nonprogressors (LTNP) [18] and elite controllers [19], respectively. Little data is available on the influence of *TNF-α *SNP on these uncommon clinical forms of HIV-1 infection [11,16].

In this study we have assessed the influence of *TNF-α *SNP on the vulnerability to HIV-1 infection and, in infected patients, on disease progression. For this purpose we analysed a cohort that we have already used for several immunogenetic studies on HIV-1 infection which contains a subset of repeatedly exposed but uninfected individuals and a carefully collected cohort of extreme long-term nonprogressors [20,21]. Given its modulating role in HIV-1-infection and disease progression reported elsewhere [22], the *CCR5Δ32 *allele was also assessed.

## Methods

### Design and setting

This was a multicenter cross-sectional population association study. All subjects were recruited from a prospectively collected cohort of almost 6000 HIV-1-infected patients treated at the HIV outpatients' clinics of the participating hospitals, which are located in an epidemiological setting in which intravenous drug use is one of the main causes of HIV-1-infection.

### Population

Two subsets of HIV-1-infected patients were studied: LTNP and typical progressors (TP). They were recruited between 2005 and 2007. Criteria for LTNP were: asymptomatic HIV-1 infection of over 16 years' duration; in the absence of any antiretroviral treatment, a stable CD4+ cell count persistently over 500 cells/μl; and a plasma HIV-1 viral load repeatedly under 5000 copies/ml [18]. LTNP were further divided into two subgroups depending on whether the plasma viral load was detectable (LTNP-DVL) or persistently undetectable. LTNP in the latter subgroup were called elite controllers (LTNP-EC) [19]. Patients were categorized as TP if they fulfilled all the following criteria: a) the HIV-1 infection had progressed to the advanced disease (that is to say class C HIV-1 disease had appeared according to the 1993 Centers for Disease Control criteria [23]), b) the plasma HIV-1 viral load was over 35,000 copies/ml and, c) the CD4+ T-cell count decreased over time and was below 350 cells/μl at least once in the first 10 years of infection. Almost all of them were on antiretroviral therapy when they enrolled. For a few patients whose date of infection was not available, we assumed that it was the midpoint between the first positive and the last negative HIV-1 blood test [24,25]. We identified 75 patients within our cohort who fulfilled the LTNP criteria. They all agreed to participate in the study, and we recruited a randomly selected group of TP (n = 109) whose age (± 5 years) and gender were comparable with the LTNP. A group of 36 repeatedly exposed but uninfected individuals (EU) was also evaluated. They were part of a cohort of individuals that we have used in other studies and whose details and characteristics we have extensively described elsewhere [26,27]. For the control group we studied a sample of healthy subjects recruited from voluntary blood donors, whose age and gender were comparable with the patients. Table 1 shows details of the study population. All subjects in our study were white Spaniards. Immigrants from other countries, including those from other European countries, and their descendents were excluded. Informed consent was obtained from each participant. The project was approved by the local ethical research committees.

**Table 1:** Demographic and clinical characteristics of the population analysed

Variable	Healthy controls (n = 203)	EU (n = 36)	HIV-1 TP# (n = 109)	HIV-1 LTNP# (n = 75)	*p *value
Male (%)	146 (72%)	22 (61%)	78 (71.5%)	50 (66.6%)	0.59*

Age (years) (mean ± SD)	44.2 ± 9.9	46.7 ± 9.2	41.8 ± 8.9	41.6 ± 7.1	0.39**

Duration of HIV-1 infection (mean ± SD)	-	-	6.2 ± 2.7	18.2 ± 1.6	< 0.0001 ##, ***

Time span between documented infection and the onset of antiretroviral treatment (mean ± SD)	-	-	4.8 ± 1.3	-	-

Plasma HIV-1 RNA (copies/ml) (median and range)	-	-	11,808 (<50->750,000)	267 (<50-4,600)	< 0.0001 ##, ****

CD4+ T-cell count (cells/μl) (median and range)	-	-	478 (6-1404)	772 (503-2080)	< 0.0001 ##,****

Exposure to HIV-1	-	22 (61%)	61 (56%)	47 (62.7%)	0.64*
Parenteral	-	14 (39%)	39 (35.8%)	22 (29.3%)	0.53*
Sexual	-	-	9 (8.2%)	6 (8%)	0.82*
Other					

• EU: Individuals repeatedly exposed to HIV-1 but uninfected

• HIV-1 TP: HIV-1-infected typical progressors

• HIV-1 LTNP: HIV-1-infected long-term non-progressors

• <50 was arbitrarily counted as 49

# The studies made of HIV-1 TP and HIV-1 LTNP were cross-sectional and the viral load and CD4 cell counts are those of the date of selection. More than 90% of HIV-1 TP were under highly active antiretroviral therapy.

## *p *value stems from the comparison between HIV-1 TP and HIV-1 LTNP.

* *p *value results of the χ^2 ^test;

** *p *value results of the ANOVA test

*** *p *value results of the Student T test

**** *p *value results of the Mann-Whitney U test

### DNA and plasma samples

Blood samples with ethylene diamine tetra-acetic acid were obtained from an antecubital vein. Five mL of whole blood was used to determine the CD4+ T-cell count, and 500 μl was used to isolate DNA with a MagNa Pure LC Instrument (Roche Diagnostics, Basel, Switzerland). Plasma for determining HIV-1 viral load was obtained by centrifugation at 3500 g for 15 minutes at 4C°.

### Laboratory methods

#### HIV-1 infection

This was diagnosed using an enzymoimmunoanalysis and confirmed by a Western-Blot test.

#### Plasma HIV-1 viral load

This was determined by the HIV Cobas Ampliprep CAP-CTMHIV-1 using the COBAS AMPLICOR system (Roche Diagnostics, Basel, Switzerland). The cutoff for undetectable viral load was 50 copies/μl.

#### Assessment of blood CD4+ T-cell count

Samples were analyzed in a flow cytometer FAC Scan (Becton Dickinson Immunocytometry Systems, San José, CA, USA). The data acquired were analyzed using the Multiset program.

### TNF-α genotype

#### *-238 TNF-α *genotype (rs 361525)

This polymorphism consists of a G→ A substitution at position -238 in the proximal promoter of the *TNF-α *gene. The primers used in PCR were: forward primer 5' AGAAGACCCCCCTCGGAACC3', modified in 3' for RFLP analysis with Msp I restriction endonuclease, and reverse primer 5' ATCTGGAGGAAGCGGTAGTG 3'. A fragment of 152 bp was amplified at a final volume of 50 μL, with 3 mM of MgCl_2_, 0.2 mM dNTPs, 0.2 μM of each primer and 1 unit of Taq polymerase. DNA was amplified for 35 cycles: 95° for 30 seconds, 55° for 30 seconds and 72° for 30 seconds. PCR products were digested with MspI and revealed a fragment of 152 bp for the A allele and two fragments of 133 and 19 bp for the G allele.

#### *-308 TNF-α *genotype (rs 1800629)

This polymorphism consists of a G→ A substitution at position -308 in the proximal promoter of the *TNF-α *gene. The primers used in the PCR were 5'AGGCAATAGGTTTTGAGGGCCAT3' and 5'TCCTCCCTGCTCCGATTCCG3'. Amplification was performed at a final volume of 50 μl containing 3 mM MgCl_2_, 0.5 mM of each nucleotide (Boehringer Mannheim™, Manheim, Germany), 0.2 μl of each oligonucleotide and 1 U of Taq polymerase (Gibco BRL). DNA was amplified for 35 cycles with denaturation at 94°C for 1 minute (min), annealing at 60°C for 1 min and extension at 72°C for 1 min. The first cycle was at 94°C for 3 min, at 60°C for 1 min and at 72°C for 1 min, followed by the 35 cycles and, finally, a cycle at 94°C for 1 min, at 60°C for 1 min and at 72°C for 5 min. The PCR products were digested at 37°C with NcoI for 24 hours, subjected to 2.5% agarose gel electrophoresis at 80 V and stained with ethidium bromide. The 107 bp band corresponded to the A allele and the set of 87 bp and 20 bp bands corresponded to the G allele.

#### *-863 TNF-α *genotype (rs 1800630)

This polymorphism consists of a C→ A substitution at position -863 in the proximal promoter of the *TNF-α *gene. The primers used in the PCR were 5'GGCTCTGAGGAATGGGTTAC3' and 5'CTACATGGCCCTGTCTTCGTTACG3'. Amplification was performed at a final volume of 25 μl containing 1 mM MgCl_2_, 0.2 mM of each nucleotide (Boehringer Mannheim™, Manheim, Germany), 1.2 μl of each oligonucleotide and 0.5 U of Taq polymerase (Gibco BRL). DNA was amplified for 35 cycles with denaturation at 94°C for 30 seconds, annealing at 62.3°C for 1 min and extension at 72°C for 2 min. The first cycle was at 94°C for 3 min, followed by the 35 cycles and, finally, an extension at 72°C for 5 min. The amplified product was digested with Bsa AI restriction enzyme (New England Biolabs) at 37°C for 24 hours, electrophoresed on 2.5% agarose gel at 80 V and stained with ethidium bromide. The 126 pb band corresponded to the C wild-type allele and the set of 103 bp and 23 pb bands corresponded to the variant A allele.

### *CCR5Δ32 *genotype

The PCR primers used were 5'GCTCTCTCCCAGGAATCATC3' and antisense 5'TTCCCGAGTAGCAGATGACC3' with annealing at 60°C. The Genbank accession number was AF031237.1. The PCR products were visualized on 2.5% agarose gel. The wild-type *CCR5 *gene led to a 174 bp fragment and the *CCR5Δ32 *variant resulted in a 142 bp fragment.

### Statistical analysis

Descriptive data were expressed as the mean ± SD or median (range) for non-parametric distributions. The differences in levels between groups in continuous variables were compared using Student's t test, the Mann-Whitney U test, or ANOVA when necessary. To evaluate the association between HIV-1 TP and the different categories of uninfected subjects for the different genotypes, the odds ratio (OR) and 95% confidence interval (95%CI) were calculated. The Hardy-Weinberg equilibrium was assessed by the chi-square goodness-of-fit test. Genotype distribution and allele frequencies in the different groups were compared by the χ2 test or Fisher's exact test when necessary. To evaluate the association of *TNF-α *genetic variants and *CCR5Δ32 *with the risk of HIV-1 infection, the differences in the distribution between HIV-1-infected, HC and EU were assessed. To evaluate the association of *TNF-α *genetic variants and *CCR5Δ32 *with disease progression in infected subjects, we compared HIV-1-infected TP vs HIV-1-infected LTNP. In the latter subset, we also compared LTNP-DVL and LTNP-EC. Analyses were performed using the SPSS/PC+ statistical package (v. 12.0 for Windows; Chicago, Illinois, USA). Haplotype frequencies were estimated by the maximum-likelihood method. Haplotype frequencies were estimated by the PAC-likelihood method [28], which takes into account the similarity of haplotypes and the fact that linkage disequilibrium decays with distance. The distributions of the different haplotypes between groups were compared by logistic regression analysis. The commonest haplotype (which we arbitrarily called haplotype H1) was taken as a reference. Haplotype analyses were performed using the PHASEv2.1 software [29,30]. A *p *value < 0.05 was considered significant.

## Results

Four hundred and twenty-three individuals were studied: 239 uninfected (203 healthy controls [HC] and 36 EU) and 184 HIV-1-infected. Of the infected individuals, most of whom had acquired HIV-1 through intravenous drug use, 109 were TP and 75 were LTNP. Of the LTNP, 20 were elite controllers. The age, gender and risk factors of acquiring HIV-1 infection in the TP and LTNP were not significantly different. Most TP (>90%) were receiving highly active antiretroviral therapy. Table 1 shows selected characteristics of the population studied. As expected, the duration of HIV-1 infection was significantly greater in LTNP than in TP. CD4+ T-cell counts were also greater and viral loads were lower.

### *TNF-α *gene polymorphisms

Tables 2 and 3 show the genotype distribution and allele frequencies of the *TNF-α-238G>A, -308G>A *and *-863 C>A *gene promoter SNP and the *CCR5Δ32 *allele for the control group and the different patient categories. The genotype distribution in control subjects, EU, HIV-1-infected, HIV-1 TP and HIV-1 LTNP fits the expected Hardy-Weinberg equilibrium for each *TNF-α *SNP.

The risk of infection showed no significant associations with the *TNF-α-238G>A *and *-863G>C *SNP; carriers of the *TNF-α-308A *genetic variant were overrepresented among uninfected individuals, but the differences did not reach statistical significance (genotype comparisons, p = 0.05; allele comparisons, p = 0.07) (Table 2).

**Table 2:** *TNF-α *and *CCR5Δ32 *genotype and allele frequencies in HC, EU and HIV-1-infected for assessment of associations with the risk of HIV-1 infection.

Genotype and allele frequencies	HC (n = 203)	EU (n = 36)	HIV-1-infected (n = 184)	*p *value *
***TNF-α-238 G>A***				
				
*GG*	147 (87.5%)	29 (80.5%)	161 (87.5%)	
*GA*	20 (12%)	6 (16.7%)	22 (12%)	0.7
*AA*	1 (0.5%)	1 (2.8%)	1 (0.5%)	
				
*GA+AA*	21 (12.5%)	7 (19.5%)	23 (12.5%)	0.5
				
Variant allele *A*	22 (6.5%)	8 (11.1%)	24 (6.5%)	0.3

***TNF-α-308G>A***				
				
*GG*	122(70.9%)	24 (67%)	148 (80.4%)	
*GA*	42(24.4%)	12 (33%)	31 (16.8%)	0.05
*AA*	8 (4.7%)	0	5 (2.8%)	
				
*GA+AA*	50 (29.1%)	12 (33%)	36 (19.6%)	0.05
				
Variant allele *A*	58 (16.9%)	12 (16.7%)	41 (11.1%)	0.07

***TNF-α-863 C>A***				
				
*CC*	116 (69.9%)	27 (75%)	137 (74.4%)	
*CA*	44 (26.5%)	8 (22%)	45 (24.5%)	0.7
*AA*	6 (3.6%)	1 (3%)	2 (1.1%)	
				
*CA+AA*	50 (30.1%)	9 (25%)	47 (25.5%)	0.6
				
Variant allele *A*	56 (16.8%)	10 (14%)	49 (13.3%)	0.4

***CCR5Δ32***				
*wt/wt*	174 (87%)	31 (86.1%)	144 (78.3%)	
*wt/Δ32*	26 (13%)	5 (13.9%)	40 (21.7%)	0.15
*Δ32/Δ32*	0	0	0	
				
*Δ32 *allele	26 (6.5%)	5 (6.9)	40 (10.9%)	0.2

• HC: healthy controls

• EU: individuals repeatedly exposed to HIV-1 but uninfected

• wt indicates wild-type allele; *Δ32*, 32 bp deletion

• Genotype and allele numbers do not match the HC studied because DNA for *TNF-α *and *CCR5 *genotyping was not available or could not be amplified in some people

* χ2 test or Fisher exact test when necessary. *p *value arises from the comparison between HC, EU and HIV-1-infected.

Both genotype and allele analyses indicated that there were no differences in the distribution of the genetic variants between LTNP and TP (Table 3). When we analysed the LTNP subset of patients and compared the elite controllers with those with detectable viral loads, we found that the *TNF-α-238A *genetic variant was significantly overrepresented in elite controllers: p = 0.02 for genotype analyses and p = 0.03 for allele analyses (Table 3). The results obtained in the full cohort for all these analyses were maintained when the individuals carrying the *CCR5Δ32 *variant allele were excluded.

**Table 3:** *TNF-α *and *CCR5Δ32 *genotype and allele frequencies in the different subsets of HIV-1-infected patients for assessment of associations with disease progression.

Genotype and allele frequencies	HIV-1 TP (n = 109)	HIV-1 LTNP (n = 75)	*p *value *	HIV-1 LNTP-DVL (n = 55)	HIV-1 LTNP-EC (n = 20)	*p *value **
***TNF-α-238G>A***						
						
*GG*	97 (90%)	64 (85.3%)		50 (90.9%)	14 (70%)	
*GA*	11 (10%)	11 (14.7%)	0.35	5 (9.1%)	6 (30%)	0.02
*AA*	1 (1%)	0		0	0	
						
*GA+AA*	12 (11%)	11 (14.7%)	0.6	5 (9.1%)	6 (30%)	0.02
						
Variant allele *A*	13 (6%)	11 (7.3%)	0.7	5 (4.5%)	6 (15%)	0.03

***TNF-α-308 G>A***						
						
*GG*	86 (78.9%)	62 (82.7%)		46 (83.6%)	16 (80%)	
*GA*	20 (18.3%)	11 (14.7%)	0.7	8 (14.5%)	3 (15%)	0.7
*AA*	3 (2.8%)	2 (2.6%)		1 (1.9%)	1 (5%)	
						
*GA+AA*	23 (21.1%)	13 (17.3%)	0.5	9 (16.4%)	4 (20%)	0.7
						
Variant allele *A*	26 (11.9%)	15 (10%)	0.6	10 (9.1%)	5 (12.5%)	0.7

***TNF-α-863 C>A***						
						
*CC*	84 (77.1%)	53 (70.6%)		37 (67.3%)	16 (80%)	
*CA*	25 (22.9%)	20 (26.6%)	0.2	17 (30.9%)	3 (15%)	0.3
*AA*	0	2 (2.8%)		1 (1.8%)	1 (5%)	
						
*CA+AA*	25 (22.9%)	22 (29.3%)	0.4	18 (32.7%)	4 (20%)	0.3
						
Variant allele *A*	25 (11.5%)	24 (16%)	0.2	19 (17.3%)	5 (12.5%)	0.4

***CCR5Δ32***						
						
*wt/wt*	88 (80.7%)	57 (76%)		39 (70.9%)	18 (90%)	
*wt/Δ32*	21 (19.3%)	18 (24%)	0.4	16 (29.1%)	2 (10%)	0.1
*Δ32/Δ32*	0	0		0	0	
						
Variant allele *Δ32*	21 (9.6%)	18 (12%)	0.5	16 (14.5%)	2 (5%)	0.1

• HIV-1 TP: HIV-1-infected typical progressors

• HIV-1 LTNP: HIV-1-infected long-term nonprogressors

• HIV-1 LTNP-DVL: HIV-1-infected long-term nonprogressors with detectable plasma viral load

• HIV-1 LTNP-EC: HIV-1-infected long-term nonprogressors with undetectable plasma viral load

• wt indicates wild-type allele; *Δ32*, 32 bp deletion

* χ2 test or Fisher exact test when necessary. *p *value arises from the comparison between HIV-1 TP and HIV-1 LTNP.

** χ2 test or Fisher exact test when necessary. *p *value arises from the comparison between HIV-1 LTNP-DVL and HIV-1 LTNP-EC.

*TNF-α *haplotype analyses indicated that there was a significant association between carriage of the haplotype 3 and the risk of infection (p = 0.04) (Table 4). No significant associations were observed with disease progression in infected individuals (Table 5). It should be noted that the EU subset could not be included in the analyses because of the low number of individuals in our cohort.

**Table 4:** *TNF-α *haplotypes and risk of HIV-1-infection.

Haplotypes	*TNF-α *SNP			n (estimated frequencies in %)			OR	OR 95CI%	*p *value *
				
	*-238 G>A*	*-308 G>A*	*-863 G>A*	Total (n = 373)	Uninfected **(n = 189)**^1^	HIV-1-Infected (n = 184)			
H1	G	G	C	246 (65.98%)	115 (61.45%)	131 (70.73%)	1.00		

H2	G	G	A	54 (14.46%)	31 (16.66%)	23 (12.51%)	1.47	0.95 - 2.30	0.09

H3	G	A	C	48 (13.07%)	29 (15.36%)	19 (10.45%)	1.65	1.04 - 2.64	0.04

H4	A	G	C	21 (5.45%)	10 (5.13%)	11 (5.99%)	1.03	0.52 - 2.04	0.94

rare	*	*	*	5 (1.04%)	-	-	1.46	0.23 - 9.47	0.69

Note: The exposed uninfected subset of patients could not be included in the analysis because of the low number of individuals available.

^1^Of 203 healthy uninfected controls individuals, only 189 had the three *TNF-α *SNPs assessed.

* *p *value for comparisons of each haplotype with a frequency greater than 1% with the most common haplotype (H1)

**Table 5:** *TNF-α *haplotypes and disease progression in HIV-1-infected patients.

Haplotypes	*TNF-α *SNP			n (estimated frequencies in %)			OR	OR 95CI%	*p *value *
				
	*-238 G>A*	*-308 G>A*	*-863 G>A*	Total (n = 184)	HIV-1 TP (n = 109)	HIV-1 LTNP (n = 75)			
H1	G	G	C	131 (70.73%)	80 (73.44%)	51 (66.70%)	1.00		

H2	G	G	A	23 (12.51%)	12 (9.90%)	11 (16.04%)	1.83	0.92 - 3.64	0.09

H3	G	A	C	19 (10.45%)	12 (9.83%)	7 (9.95%)	1.20	0.60 - 2.41	0.61

H4	A	G	C	11 (5.99%)	5 (4.25%)	6 (7.30%)	1.84	0.68 - 4.95	0.23

rare	*	*	*	0 (0.32%)	-	-	1.00	-	1.00

* *p *value for comparisons of each haplotype with a frequency greater than 1% with the most common haplotype (H1)

HIV-1 TP: HIV-1-infected typical progressors

HIV-1 LTNP: HIV-1-infected long-term non-progressors

### *CCR5 Δ32*

There were no significant differences in the prevalence of the *CCR5Δ32 *genetic variant allele among the different categories of patients assessed (Tables 2 and 3). *CCR5Δ32 *homozygosity was not detected.

## Discussion

In this cohort of Caucasian Spaniards, we found that *TNF-α *gene polymorphism may be involved in vulnerability to HIV-1 infection. In infected patients, none of the *TNF-α *genetic variants assessed influences disease progression, but the *TNF-α-238G>A *SNP may modulate viremia control in LTNP. Carriage of the *CCR5Δ32 *variant allele does not influence the risk of infection or disease progression.

To explain the variable interindividual vulnerability to HIV-1 infection, we sought a genetically-driven host susceptibility. Several candidate genes were checked, one of which was *TNF-α *because of its robust role in initial response to infection influences both innate and acquired immunity [1,3,31,32]. Our data indicate that none of the individual polymorphisms assessed influence vulnerability to HIV-1 infection, but haplotype analyses suggest that the combination of some genetic variants within the *TNF-α *gene may modulate the risk of infection. These results agree with some reports that showed an association between *TNF-α *SNP and the risk of infection [10,12]; other investigations, however, observed no significant associations [11,13-15]. The discrepancy between our findings and those reported previously may be because of the low number of patients assessed in some studies [15], which often provides unstable data [33], or because the different ethnicities of the people assessed in others [13,14] may have led to genuine population differences [34].

Other reports have investigated the involvement of *TNF-α *genetic variants in a variety of immunogenetic events that affect HIV-1 disease progression. Some studies have related the risk of progression [10-12,16,17] and/or of developing a diversity of AIDS-related events [35-41] with carriage of some *TNF-α *genetic variants while others, like ours, have failed to find any significant association [14,15]. In this respect, our study suggests that there are no significant associations between *TNF-α *genetic variants and disease progression. To assess this issue we used a cohort of TP as controls and a subset of LTNP of over 16 years' duration as cases. This extreme LTNP phenotype was expressly chosen to prevent groups from superposing. Of particular note, however, was that within the LTNP group we showed a significant association between carriage of the *TNF-α-238A *variant allele and the spontaneous "elite controller" phenotype. This replicates what was reported in an early study [16] but, since this finding is supported by a very small number of patients it should be further replicated in larger series of this uncommon subset of HIV-1-infected patients.

For *CCR5Δ32*, we found that there is no signal for non progression. This is in agreement with *CCR5Δ32 *having a primary effect against rapid progression as pointed out by the GRIV study [42,43]. We acknowledge, however, that in order to see the effect of *CCR5Δ32*, the use of a larger cohort and/or a Kaplan-Meier plot of patients' evolution since seroconversion under various endpoints could have been useful.

Our study has some limitations. First, the cross sectional nature of the design provides associations, not causality. Second, the comparison of the LTNP with the TP subsets is rather limited since it involves long-term non progression and not simple progression, and additional analyses with Kaplan-Meier curves (with various endpoints such as death or AIDS 1993 criteria) could lead to different results. Finally, some of the subsets assessed were small, particularly the exposed uninfected and elite controllers. This means that our analyses of these subsets may be too underpowered to detect other significant associations.

## Conclusions

In summary, in a cohort of Caucasian Spaniards, polymorphism within the *TNF-α *gene may be associated with vulnerability to HIV-1 infection. In infected patients, none of the *TNF-α *genetic variants assessed influences disease progression but the *TNF-α-238G>A *SNP may modulate the elite controller status. The *CCR5Δ32 *variant allele influences neither the risk of infection nor disease progression.

## Competing interests

The authors declare that they have no competing interests.

## Authors' contributions

SV, FG, PD, CAV, EP, JMG, CR and FV designed the study. SV, JP, CV, MG, AL and MT collected and analysed the data. MB, MP and CA performed the genetic studies. MO and MLD performed the statistical analyses. SV, MO, FG, PD, CAV, EP, JMG, CR and FV drafted the manuscript. All authors have read and approved the manuscript.

## Pre-publication history

The pre-publication history for this paper can be accessed here:

http://www.biomedcentral.com/1471-2350/11/63/prepub
